# Dr. Moses T. Babcock

**Published:** 1902-05

**Authors:** 


Dr. Moses T. Babcock, of Hammondsport, N. Y., where he had practised his profession for about 50 years, died suddenly at his home in that village, March 31, 1902. He served as assistant-surgeon of the 141st regiment, N.Y. Infantry, during the civil war and was greatly respected in the community where he lived so long.

